# Genetic testing enables a precision medicine approach for nephrolithiasis and nephrocalcinosis in pediatrics: a single-center cohort

**DOI:** 10.1007/s00438-022-01897-z

**Published:** 2022-05-25

**Authors:** Lin Huang, Chang Qi, Gaohong Zhu, Juanjuan Ding, Li Yuan, Jie Sun, Xuelian He, Xiaowen Wang

**Affiliations:** 1 Department of Nephrology, Wuhan Children’s Hospital (Wuhan Maternal and Child Healthcare Hospital), Tongji Medical College, Huazhong University of Science and Technology, Wuhan, 430000 China; 2 Department of Ultrasonic Imaging, Wuhan Children’s Hospital (Wuhan Maternal and Child Healthcare Hospital), Tongji Medical College, Huazhong University of Science and Technology, Wuhan, 430000 China; 3 Department of Central Laboratory, Wuhan Children’s Hospital (Wuhan Maternal and Child Healthcare Hospital), Tongji Medical College, Huazhong University of Science and Technology, 100 Hong Kong Road, Wuhan, 430014 Hubei China

**Keywords:** Nephrolithiasis, Nephrocalcinosis, Hereditary etiology, Genetic testing, Pediatric

## Abstract

**Objective:**

Hereditary factors are the main cause of pediatric nephrolithiasis (NL)/nephrocalcinosis (NC). We summarized the genotype–phenotype correlation of hereditary NL/NC in our center, to evaluate the role of genetic testing in early diagnosis.

**Methods:**

The clinical data of 32 NL/NC cases, which were suspected to have an inherited basis, were retrospectively analyzed from May 2017 to August 2020. The trio-whole exome sequencing was used as the main approach for genetic testing, variants were confirmed by Sanger sequencing, and pathogenicity analysis according to protein function was predicted with custom-developed software.

**Results:**

Causative monogenic mutations were detected in 24 of 32 NL/NC patients, and copy number variation was detected in one patient. A summary of manifestations in patients with inherited diseases revealed a significant degree of growth retardation, increased urinary excretion of the low-molecular weight protein, hypercalciuria, electrolyte imbalances, and young age of onset to be common in heredity disease. In addition, some patients had abnormal renal function (3 ppm 25). The most frequent pathology identified was distal renal tubular acidosis (with inclusion of *SLC4A1*, *ATP6V1B1*, and *ATP6VOA4* genes), followed by Dent disease (*CLCN5* and *OCRL1* genes), primary hyperoxaluria (PH) (*AGXT* and *HOGA1* genes) and Kabuki syndrome (*KMT2D* gene), which was more likely to present as NC or recurrent stone and having a higher correlation with a specific biochemical phenotype and extrarenal phenotype.

**Conclusion:**

The etiology of NL/NC is heterogeneous. This study explored in depth the relationship between phenotype and genotype in 32 patients, and confirmed that genetic testing and clinical phenotype evaluation enable the precision medicine approach to treating patients.

**Supplementary Information:**

The online version contains supplementary material available at 10.1007/s00438-022-01897-z.

## Introduction

Epidemiological data involving numerous countries around the world show that the incidence of nephrolithiasis (NL) is 1–19%, especially in Asian countries (Khan et al. 2016). Recent data show that the incidence in children is increasing at about 10% a year (Hernandez et al. 2015; Routh et al. 2010). The young age of onset and painful recurrent disorder associated with urethral obstruction and recurrent urinary tract infections (UTI) were thought to be associated with a higher risk of acute kidney injury (AKI), chronic kidney disease (CKD), and mineral bone disease (MBD) (Bonzo and Tasian 2017; Sakhaee et al. 2011; Tang and Lieske 2014). Nephrocalcinosis (NC) is defined as the generalized deposition of calcium oxalate (CaOx) or calcium phosphate (CaPi) in the kidney. Due to similarity in clinical manifestations between NL and NC, the latter is frequently misdiagnosed, and it is often associated with a loss of renal function (Shavit et al. 2015).

The etiology of NL/NC is multifactorial and is linked to genetics, systemic diseases (inflammatory bowel disease, parathyroid dysfunction), metabolic factors, renal structural abnormalities, and recurrent urinary tract infections (Khan et al. 2016). It is distinguishable from the common metabolic factors such as diabetes, hypertension, and obesity in adults. Hypercalciuria is the most common metabolic abnormality detected in children with NL or NC, and is especially highly significant for recurrent NL or progressive NC (Gürgöze and Sarı 2011; Saitz et al. 2017; Spivacow et al. 2008).

A previous analysis of 268 NL/NC patients and 134 pediatric NL/NC patients showed that monogenic causative mutations in one of more than 30 known NL/NC genes could be detected in 11.4% of adult individuals, 16.7–20.8% of individuals < 18 years of age (Amar et al. 2019; Braun et al. 2016; Daga et al. 2018; Halbritter et al. 2015), and 0.4% of early-onset CKD diagnoses (Vivante and Hildebrandt 2016). Numerous genetic disorders lead to NC, with or without kidney stones, including Dent disease, Lowe syndrome, Bartter syndrome, and distal renal tubular acidosis (dRTA), and patients with primary hyperoxaluria (PH). CKD has been frequently observed in patients with recurrent NL or progressive NC with a long history of the disease. The search for pathogenetic genes for patients who are suspected to have hereditary NL/NC can help us to make precision genetic diagnoses as well as provide targeted medicine interventions.

This article summarizes the genotype–phenotype correlation of hereditary NL/NC in a single center. It is aimed to assess the benefits of genetic testing in the early diagnosis of hereditary NL/NC and discusses the strategy of genetic testing to relieve the health care costs.

## Materials and methods

### Cohort

We performed a retrospective review of a total of 32 patients from unrelated Chinese families with a diagnosis of hereditary NL/NC in our center from May 2017 to August 2020. Included are 21 males and 11 females, ranging in age from 3 months to 14 years (Supplementary Tables 1 and 2). The study protocol was approved by the Ethics Committee of Wuhan Children’s Hospital (Wuhan, China). Informed consent was obtained from all legal guardians included in our study. All of the patients were diagnosed with NL or (and) NC by ultrasound or computed tomography (CT) (2 cases), and underwent metabolic evaluation, including measurement of electrolyte levels (calcium, magnesium, potassium, sodium, chlorine, and phosphorous), creatinine levels to evaluate renal function, urine routine, urinary calcium/creatinine ratio or urinary calcium levels in 24-hours urine samples and demographic data. When necessary, parathyroid hormone (PTH) and vitamin D levels were measured, and bone X-ray and stone analysis were implemented.

### Gene sequencing

The blood samples of the patients and their parents were obtained. The trio-whole exome sequencing was used as the main genetic testing, variants were confirmed by Sanger sequencing and pathogenicity analysis by protein function was predicted with custom-developed software. Two patients received copy number variation (CNV) analysis because of a facial dysmorphism and psychomotor retardation.

Samples were sequenced to at least 2.5 GB, yielding paired 250 nucleotide reads. Genomic DNA was isolated from blood lymphocytes and subjected to exome capture using IDT the xGen Exome Research Panel v2.0, according to manufacturers’ protocols (Illumina, San Diego, CA, USA). The pooled libraries were sequenced on an Illumina HiSeq 2000 sequencing platform. A minimum 50× coverage was achieved in our cohort, and target sequencing coverage was not less than 99%. Sequence reads were mapped to the human reference genome assembly (37/hg19 https://www.ncbi.nlm.nih.gov/genome), assessing the frequency of a variant by searching publicly available population databases (1000 Genomes Project http://browser.1000genomes.org/, Exome Aggregation Consortium ExAC http://exac.broadinstitute.org/), and determining the pathogenicity of the variant through the database including DECIPHER (http://decipher.sanger.ac.uk/) and ClinVar (http://www.ncbi.nlm.nih.gov/clinvar). Variants were filtered by frequency and variant category and confined with a minor allele frequency (MAF) of 1% or less in the Online Mendelian Inheritance in Man (OMIM, https://omim.org/). Protein function was predicted with software SIFT (Sorting Intolerant from Tolerant, http://sift.jcvi.org) and PolyPhen2 (Polymorphism Phenotyping v2, http://genetics.bwh.harvard.edu/pph2). The American College of Medical Genetics and Genomics guidelines (ACMG) for variant classification was applied to classify the pathogenicity, and all variants were confirmed by Sanger sequencing.

We performed CNV analysis on whole-genome sequencing (WGS) data. Total sequencing depth was about 0.2–0.6×, and the detection sensitivity was the amplification or deletion of DNA in the size of 0.1 Mb. We performed the pathogenicity of variants using DGA (Database of Genomic Variants, http://dgv.tcag.ca/dgv/app/home).

Analysis of mutations was performed by a team of clinician–scientists and geneticists with domain expertise in inherited kidney diseases and bioinformaticians. Pathogenic and likely pathogenic variants were identified as pathogenic variation, and uncertain pathogenic variants were identified as variants of unknown significance (VUS).

### Statistical tests

The age variable did not conform to the normal distribution using the K–S normality test and was expressed by median and quartile in the statistical description. A chi-squared test or Fisher’s exact test was used for comparative analysis of qualitative variables of the incidence of NL or NC between different groups. In the case of non-normal distribution, the Wilcoxon test was used for comparison of quantitative variables. A *P* value of < 0.05 indicated statistical significance, based on a Fisher’s exact test compared to controls and the analysis of data was performed through SPSS software (IBM SPSS Statistics 22).

## Results

### Cohort characteristics and clinical features

**Fig. 1:**
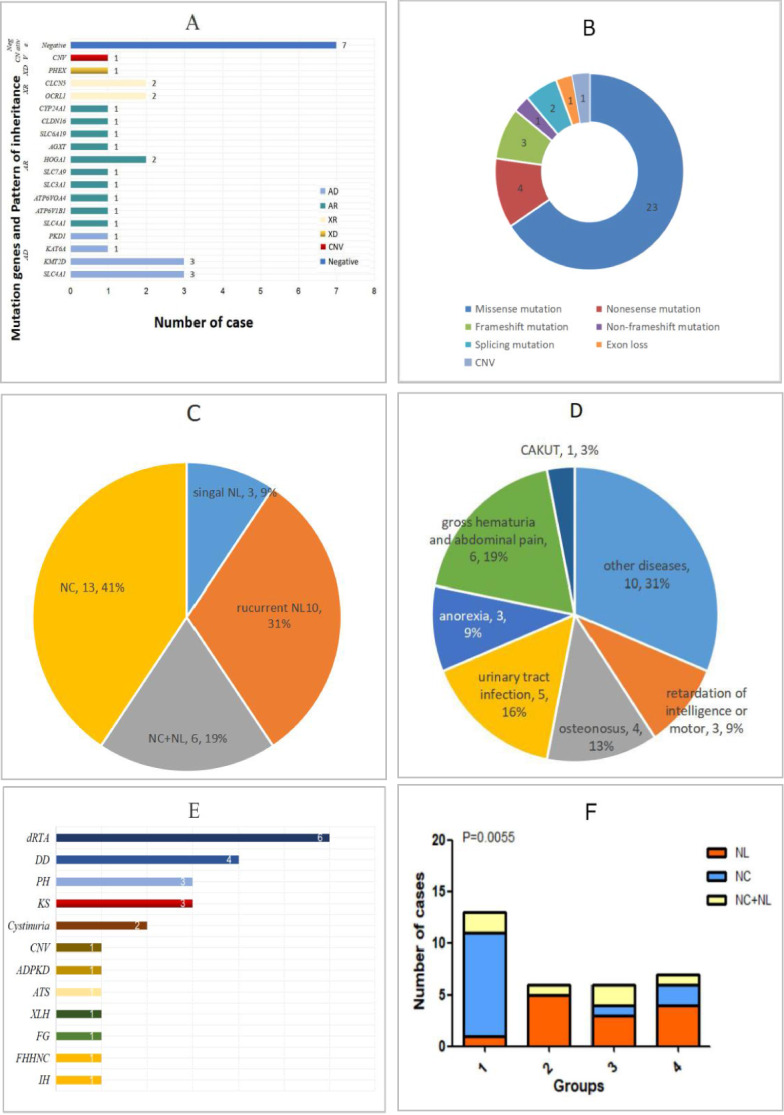
Molecular diagnostic results and clinical features in patients with NL/NC. **A** A summary of testing results is illustrated. AD and AR are the most common genetic mode, and the most frequent variant genes are *SLC4A1* and *KMT2D.* Among them, *SLC4A1* had two genetic patterns of AD and AR. **B** A total of 35 variant mutations have been detected, including 23 missense mutations, 4 nonsense mutations, 1 non-frameshift mutation, three frameshift mutations, two splicing mutations, and one exon loss. **C** A summary of manifestations revealed that there were 13 cases had isolated NC, 19 cases had NL, among them, 10 had multiple stones, 6 had NL merges NC, and 3 had a single stone. **D** Gross hematuria, abdominal pain, and recurrent infection are more likely to exist if symptoms are present. However, most of the patients are asymptomatic; presentations leading to diagnosis include osteonosus, retardation of intelligence or motor and anorexia. **E** The two most frequent diseases were dRTA and Kabuki syndrome, followed by Dent disease and PH. **F** The patients with failed urinary acidification and hypercalciuria (group 1) were more likely to show NC (*P* = 0.0009 for chi-squared test), and the patients with hyperuricosuria, cystinuria, and hyperglycinuria (group 2) were more likely to have NL (*P* = 0.0289). There was no statistical difference in the proportion of NL in other genes (group 3) and gene negative group (group 4). (*P* = 0.6664, *P* = 0.4012). NL plus NC are marked in yellow; NC is marked in blue; NL is marked in yellow

A total of 32 patients with a diagnosis of hereditary NL/NC in our center received genetic testing and underwent metabolic evaluation. Included were 21 males and 11 females ranging in age from 3 months to 14 years, with 15 patients under the age of 2 and a median of 2.54 years (0.88 years, 4.81 years). The clinical characteristics are presented in Supplementary Tables 1 and 2. The *SLC4A1* and *KMT2D* were the most frequent variant genes, and the missense variation was the most among all of the detected types (Fig. 1A, Fig. 1B). 

There were 13 cases identified with isolated NC, and 19 cases with NL. Among these, ten had multiple stones, six had NL merged with NC, and three had a single stone (Fig. 1C). A significant degree of growth retardation, increased urinary excretion of low-molecular weight protein (LMWP), hypercalciuria, experienced electrolyte imbalances, and young age of onset were common in our cohort, especially for patients with confirmed molecular diagnosis. Gross hematuria, abdominal pain and recurrent infection occurred in symptomatic patients. However, the majority of patients were asymptomatic; presentations leading to diagnosis include osteonosus, retardation of motor skills or intelligence, and anorexia (Fig. 1D).

### Genetic analysis

By whole exome sequencing (WES) or WES plus CNV, we identified a genetic diagnosis in 78.1% of cases (25 ppm 32), with 24 cases detected with monogenic disorders and 1 case with deletion of the genome fragment. The genotypic characteristics are reflected in Supplemental Table 3. Most of the patients resulted in monogenic hereditary tubulopathies with 16 monogenes. Causative mutations or likely causative mutations were detected for known NL/NC in 19 of 32 patients, and five causative mutations were detected for other genetic abnormalities and there was detection of one with copy number variation. A summary of testing results is provided in Fig. 1A and Table 1. Among them, one case was from consanguineous families, the mothers of two cases had a history of obstetric abnormality, and three cases had a family history of NL/NC. Among patients without a confirmed pathogenic mutation, five patients had a family history of stones.

**Table 1:** Cohort characteristics of 32 patients with NL and (or) NC in our center

	Variants mutation		Family history	Consanguinity	CKD^a^	Confirmed clinical diagnosis	Confirmed molecular diagnosis	Unconfirmed diagnosis
	Positive	Negative						
NL	9 (69.2%)	4 (30.7%)	3	0	1	1	9	4
NC	11 (84.6%)	2 (15.4%)	4	1	2	6	11	2
NL + NC	5 (83.3%)	1 (17.6%)	1	0	1	1	5	1
Total	25 (78.1%)	7 (21.9%)	7	1	4	8 (25%)	25 (78.1%)	7 (21.9%)

*CKD* chronic kidney disease, *AR* autosomal recessive inheritance, *CNV* chromosome copy number variation

^**a**^Note that, CKD is an uncommon complication, one patient progressed to CKD in the presence of NL, with an unconfirmed diagnosis. Two patients progressed to CKD in the presence of NC. One patient progressed to CKD in the presence of NL merged with NC. These patients have confirmed molecular diagnosis

We noted that patients < 2 years old (13 cases out of 25, 52%) with confirmed molecular diagnoses showed as main clinical features significant growth retardation (20 cases out of 25, 80%), psychomotor retardation (9 cases out of 25, 36%), hypercalciuria (8 cases out of 25, 32%), LMWP (13 cases out of 25, 52%) and electrolyte disturbances (9 cases out of 25, 36%) (Supplemental Table 1). In addition, three patients (12%) had abnormal renal function, with the estimated glomerular filtration rate ranging from 60 to 90 ml/min/1.73 m^2^, equivalent to stage two chronic kidney disease. The proportion of patients with a negative molecular diagnosis was 14.2% (1 out of 7) (Supplemental Table 2, Table 1).

In patients with monogenic disorders, a total of 16 variant genes were detected, including 23 missense mutations, four nonsense mutations, one non-frameshift mutation, three frameshift mutations, two splicing mutations and one exon loss (Fig. 1B). 23 of 33 mutations (69.7%) were novel. Patterns of inheritance included the autosomal dominant (AD), autosomal recessive (AR), X-linked dominant (XLD), and X-linked recessive (XLR). AD and AR were the most common pattern of inheritance (Fig. 1A), and the most frequent variant genes were *SLC4A1* and *KMT2D*, which can, respectively, lead to the two most frequent diseases: dRTA (with inclusion of *SLC4A1*, *ATP6V1B1*, and *ATP6VOA4* genes) and Kabuki syndrome. Dent disease (with inclusion of *CLCN5* and *OCRL1* genes) and primary hyperoxaluria (PH) (with inclusion of *AGXT* and *HOGA1* genes) were identified with high frequency (Table 2, Fig. 1E). These patients mainly had NC or recurrent stone.

**Table 2:** The genotype–phenotype correlation in 25 patients with a known genetic disease

Genetic diagnosis	Number of cases	Sex	Onset-age	Genetic testing	Phenotype	
					Renal	Extrarenal
Genes of failed urinary acidification and hypercalciuria						
Dent disease	4					
N1		M	2 years 2 months	*OCRL1* c.556 A > T p.K186X, 716	NC hypercalciuria LMWP	Growth and psychomotor retardation
N2		M	3 years 3 months	*OCRL1* c.218 T > A p.L73X, 829	NC Hypercalciuria LMWP	Growth retardation
N3		M	4 years 11 months	*CLCN5* c.478 T > C p.C160R	NL + NC Hypercalciuria LMWP	Growth retardation
N4		M	14 years	*CLCN5* c.2131 T > C p.C711R	NC hypercalciuria LMWP	-
Distal renal tubular acidosis	6					
N5		M	12.5 years	*SLC4A1* c.2102 G > A p.G701D	NC CKD2 LMWP	Growth retardation, knock knees, hypokalemia
N6		F	9 months	*SLC4A1* c.1765 C > T p.R589C	NC	Growth retardation, hypokalemia
N7		M	6 years	*SLC4A1* c.1766 G > A p.R589H	NL hypercalciuria LMWP	Growth retardation, knock knees, hypokalemia
N8		F	1 years 8 months	*ATP6V1B1* c.1153 C > A p.P385T c.806C > T p.P269L	NC	Growth retardation, hypokalemia, LMWP
N9		F	3 months	*ATP6VOA4* c.1899 C > A p.Y633X, 208 hom	NC	Growth retardation, hypokalemia, ASD
N10		M	3 years 11 months	*SLC4A1* c.1765 C > T p.R589C	NL + NC hypercalciuria LMWP	Growth retardation, hypokalemia
X-1inked dominant hypophosphate	1					
N11		F	3 years 9 months	*PHEX* c.1735 G > A p.G579R	NC	Growth retardation, Bow legs, psychomotor retardation, hypophosphatemia
Familial hypomagnesaemia with hypercalciuria and nephrocalcinosis	1					
N12		F	2 years 11 months	*CLDN16* loss1 (exon: 3) c.427 + 5 (IVS2) G > A	NC	Hypomagnesemia
Infantile hypercalcaemia	1					
N13		F	11 months	*CYP24A1* c.1310 C > A p.P437H hom	NC CKD2 LMWP	Growth and psychomotor retardation, hypercalcemia, muscle weakness
Genes of hyperuricosuria, cystinuria, hyperglycinuria						
Cystinuria	2					
N14		M	10 months	*SLC3A1* c.541C > T p.R181W c.850 G > C p.D284H c.1173 C > G p.D391E	NL, l-cystine stone	Growth retardation, ASD
N15		M	6 years 7 months	*SLC7A9* c.376 G > A p.A126T c.149 T > G p.V50G	NL, l-cystine stone	Growth retardation
Primary hyperoxaluria	3					
N16 (PH1)		M	4 months	*AGXT* c.25 _ c.26 insC p.T9Tfs * 159 c.121 G > A p.G41R	NL + NC, increased excretion of oxalic acid	Renal rickets
N17 (PH3)		M	11 months	*HOGA1* c.834 + 1 (IVS6) G > T c.356 T > G p.V119G	NL, CaOx stone	Growth retardation
N18 (PH3)		M	13 months	*HOGA1* c.103 A > G p.I35V c.845 G > A p.R282H	NL, CaOx stone	Growth retardation
Familial glycosuria	1					
N19		M	6 months	*SLC6A19* c.284 G > Cp.R95P c.461 C > T p.P154L	NL	Psychomotor retardation, ASD
Other genes						
Kabuki syndrome	3					
N20		M	9 months	*KMT2D* c.15113_c.15115 del AGG p.E5038_G5039delinsG	NL hypercalciuria	Growth and psychomotor retardation, facial dysmorphism, ASD
N21		M	1 years 8 months	*KMT2D* c.6595 delT p.Y2199fs	NL	Growth and psychomotor retardation, facial dysmorphism, ASD + VSD + PH
N22		M	8 years	*KMT2D* C.15686 dupG P.C5230Lfs*5	NC hypercalciuria LMWP	Growth and psychomotor retardation, facial dysmorphism, VSD
Arboleda-Tham syndrome	1					
N23		M	3 months	*KAT6A* c.3070 C > T p.R1024X, 981	NL signal hypercalciuria	Growth and psychomotor retardation, facial dysmorphism + ASD
Autosomal dominant polycystic kidney disease	1					
N24		M	8 years	*PKD1* c.9806 G > A p.R3269Q	NL + NC	Renal cyst + ASD
CNV	1					
N25		M	3 months	CNV chr10: 130378377–135427935 q26.2–q26.3	NL + NC CKD2	Growth and psychomotor retardation, VUR + NB/facial dysmorphism

Normal urinary values in spot urine samples and normal urinary values in 24-h urine collection: urine calcium-to-creatinine ratio: < 7 months < 0.86, 7–18 months < 0.60, 19 months–6 years < 0.42, > 6 years < 0.20 (mg/mg) or 24-h urinary calcium < 4 mg/kg/24 h. *F* female, *M* male, *LMWP* low-molecular weight protein (the elevated levels of urinary β2-microglobulin and α1-microglobulin), *ASD* atrial septal defect, *VSD* ventricular septal defect, *PH* pulmonary hypertension, *CKD* chronic kidney disease, *VUR* vesicoureteric reflux, *NB* neurogenic bladder, *CaOx* analysis of stone composition suggests calcium oxalate stone

### Genotype–phenotype correlation

#### Genes that participated in failure of urinary acidification and hypercalciuria

Of note, confirmed clinical diagnosis of NL/NC was found to be associated with certain diseases, including dRTA, Dent disease, X-linked hypophosphatemia (XLH), infantile hypercalcaemia, and familial hypomagnesaemia with hypercalciuria and nephrocalcinosis (FHHNC) (Supplemental Table 3). In all patients, the urinary excretion of LMWP was significantly increased (three cases). There was also a significant degree of growth retardation (11 out of 13 cases), with patients more likely to present with NC (*P* = 0.0009 for chi-squared test) (Fig. 1F).

In the present study, six patients with a clinical diagnosis of dRTA presented with pathogenic variants in the *SLC4A1*, *ATP6V1B1*, *ATP6VOA4* genes. Four patients had causative mutations in the *CLCN5*, *OCRL* genes, which are associated with Dent disease, and a heterozygous mutation of the *PHEX* gene was detected in a patient diagnosed with XLH. A homozygous mutation of the *CYP24A1* gene was detected in one patient and facilitated a diagnosis of infantile hypercalcaemia. Compound heterozygous mutations of the *CLDN16* gene were detected in a patient clinically diagnosed with FHHNC.

Pathogenic variants were found in all six patients with dRTA that could be clinically diagnosed due to the phenotype of normal serum anion gap metabolic acidosis, hypokalemia, and growth retardation. Most of these patients had NC or NC plus NL, and only one patient had recurrent stones. Of the four patients carrying pathogenic variants in the *SLC4A1* gene, three had AD inheritance, and one had AR inheritance. One patient has compound heterozygous mutations in the *ATP6V1B1* gene, and another patient had compound heterozygous mutations in the *ATP6V0A4* gene.

Hypercalciuria and LMWP were noted in all four patients with Dent disease, two were Dent-1 caused by *CLCN5* mutation, and two were Dent-2 caused by the *OCRL1* mutation. The patients with Dent-2 were diagnosed at the younger age of onset. Two of the patients had nonsense mutations p.K186X and p.L73X in *OCRL1* gene, and the growth and development lag were more prominent than the two patients with missense mutations p.C160R and p.C711R in *CLCN5* gene. In addition, the age of onset was younger.

XLH, infantile hypercalcaemia, and FHHNC have characteristic biochemical phenotypes. All three patients had a young age of onset. The XLH patient had bowed legs, and the patient with infantile hypercalcemia had renal calcification and loss of renal function.

#### Hyperuricosuria, cystinuria, and hyperglycinuria genes

Recurrent stones were seen in most patients with abnormal urinary oxalate, cysteine, and excretion of amino acids. These patients were finally diagnosed with cystinuria, primary hyperoxaluria (PH) and hyperglycinuria (Table 2). We diagnosed three cases with PH, two cases with cystinuria, and one case with familial renal glycosuria. These patients have a relatively young age of onset, with a median age of 10.5 months (5.5 months, 29.5 months). While there was no obvious growth retardation, LMWP or extra-renal manifestations, most had recurrent renal stones (*P* = 0.0289) (Fig. 1F). NC is a frequently observed feature that was overlooked in these patients.

Pathogenic variants of the *HOGA1* gene were found in two patients, diagnosed with PH type-III (PH3). Stone analysis suggested calcium oxalate stone. It is worth noting that one patient underwent minimally invasive surgery to remove stones causing obstruction. The age of the patient diagnosed with PH type-I (PH1) was 4 years. A frameshift mutation p.T9Tfs * 159 and a missense mutation p.G41R were detected in this patient. The renal function of the patient was normal at the time of diagnosis, and vitamin B6 treatment was implemented as he presented with signs of rickets. A stone composition analysis was unable to be obtained, however, a 24-h urinalysis revealed a significant increase in oxalic acid levels.

Two patients diagnosed with cystinuria were tested for the genetic mutations *SLC3A1* and *SLC7A9*, respectively. The compositional analysis of the stones indicated l-cysteine. The two patients were diagnosed under the age of two, and no typical staghorn calculi were observed. Following an adequate water intake and oral treatment with potassium citrate, the size of stones did not increase in the course of the next 2 years.

#### Other genes and CNV

Interestingly, in our cohort, in addition to the currently known monogenic disorders, two genes and one CNV were not previously described as causing NL/NC were detected that are involved in epigenetic modifications or could cause mental retardation. Included were three cases with Kabuki syndrome caused by the *KMT2D* gene, one case with the *KAT6A* gene, and one case with a 5.05-Mb draft genome sequence at chr10:130378377–135427935 q26.2–q26.3. The median age for this type of patient was 9 months (3 months, 58 months). Special manifestations, including facial dysmorphism, psychomotor retardation, CAKUT and congenital anomalies of other organs (such as the heart and hip joint), were common clinical phenotypes in these patients (Table 2, Supplemental Table 3).

Three patients showed long palpebral fissures, eversion of the lower lateral eyelid, arched eyebrows with sparse or dispersed lateral one third, depressed nasal tip, and large, prominent ears, combined with mild mental retardation and congenital heart disease. One case had congenital hip dislocation, and kidney stones were found via ultrasound in all of these patients. The patient with pathogenic variation of the *KAT6A* gene presented with high-arch, small-mandible, abnormal auricle, adduction of the thumb, and psychomotor retardation. Although no pathogenic variant was detected by clinical exome sequencing in one patient who had special facial features, however, a 5.05-Mb microdeletion was found. In this patient, we detected psychomotor retardation and CAKUT and congenital heart disease. Voiding cystourethrogram (VCUG) indicated grade IV right-sided vesicoureteral reflux (VUR), and the patient was suspected to have neurogenic bladder because of the changes in bladder morphology. Acute renal failure occurred due to UTI at the age of 3 months, and the renal function could not completely recover to the normal level.

There was one patient whose ultrasound examination revealed NC and NL. He was suspected to have bilateral renal microcysts, and he was later diagnosed with autosomal dominant polycystic kidney disease (ADPKD). According to the patient’s age at the time of diagnosis, no typical renal cysts were noted.

### Unconfirmed genetic diagnosis

As described in Supplemental Table 2, there were seven patients who were suspected of having hereditary NL/NC with unconfirmed clinical and molecular diagnosis, including four cases with NL, two cases with NC, and one case with NL plus NC. Growth retardation (two cases, 28.5%) and motor retardation (one case, 14.2%) were observed, however, LMWP, experienced electrolyte imbalances and CAKUT were not detected.

## Discussion

In recent years, the incidence of NL/NC in children has shown an upward trend, with a younger age of onset. Children have a high risk of recurrent, inherited NL or NC, more often associated with impaired kidney function (Zisman et al. 2015).

More than 30 genes have been reported to be associated with the monogenic forms of NL/NC. The ability to detect the causative mutation(s) in a monogenic disease gene is of great diagnostic and potentially therapeutic importance, as there is an almost deterministic cause–effect relationship in monogenic disease (Dhayat et al. 2016; Sayer 2017). Genetic studies have revealed that the following have important roles in the etiology of kidney stones: transporters and channels; the calcium-sensing receptor signaling pathway; the metabolic pathways for vitamin D; oxalate, cysteine, amino acids; purines and uric acid) (Howles and Thakker 2020). The above-mentioned abnormalities are related to monogenic disorders.

A number of Chinese scholars reported the genetic spectrum of Chinese children with renal disease via establishing the multicenter registration system (Chinese Children Genetic Kidney Disease Database, CCGKDD). According to their findings, the diagnostic yield was 62.3% presenting with renal tubular disease or NC/NL (Rao et al. 2019). These results are similar to the incidence achieved in our center for NL/NC. This similarity may be attributed to the screening criteria adapted for genetics.

In our study of children with suspected hereditary NL/NC, most had hereditary diseases with NC or recurrent stone. A summary of manifestations revealed a significant degree of growth retardation, increased urinary excretion of low-molecular weight protein, hypercalciuria, experienced electrolyte imbalances, and young age of onset to be common. These findings suggest that the significant degree of growth retardation and onset at less than 2 years of age may be a high-risk factor for etiology of NL/NC.

Diseases predominant in our study included dRTA, Dent disease, XLH, infantile hypercalcaemia, and FHHNC. The clinical diagnosis can be supported and confirmed by the clinical phenotype, however, genetic testing can help to identify molecular diagnosis. This information can be useful in determining the severity and prognosis of the disease, and can help to guide treatment and provide patients with genetic counseling.

Several clinical features, such as normal serum anion gap metabolic acidosis, renal stone, osteomalacia, growth retardation, and rickets, were recognized to be characteristic of, and diagnostic of, dRTA. To date, studies have confirmed that *SLC4A1*, *ATP6V1B1*, and *ATP6V0A4* are the main causative genes of dRTA (Lopez-Garcia et al. 2019; Palazzo et al. 2017). Patients with *SLC4A1* gene variation can inherit in an AD/AR manner, with or without deafness. In comparison, *ATP6V1B1* and *ATP6V0A4* genes have only AR genetic pattern, which is more likely to be combined with sensorineural hearing loss (SNHL) (Vargas-Poussou et al. 2006). There are regional differences in the genetic variants of *SLC4A1* gene: Caucasians are mainly reported with AD inheritance, while AD inheritance and AR inheritance were mainly reported in Southeast Asia (especially in Thailand), China, Korea, and Japan (Alonso-Varela et al. 2018; Park et al. 2018).

The *SLC4A1* was noted as the main gene in patients with dRTA in our center. Four patients carried pathogenic variants in the *SLC4A1* gene, three had AD inheritance, and one had AR inheritance. As reported in the literature, patients with inherited AD had a less severe clinical phenotype than those with AR, and the onset was older. The patient who was identified with a homozygous mutation of p.G701D was the oldest and progressed to CKD2. Neither of the two patients with the *ATP6V1B1* and *ATP6V0A4* genes variants had abnormal hearing at diagnosis. It is highly essential to follow-up on factors influencing hearing.

For Dent disease, it has been reported that 30–80% of male patients may develop end stage renal disease (ESRD) between the ages of 30–50 years. However, in our study, all four patients showed normal renal function and were closely monitored in the follow-up period (Devuyst and Thakker 2010). The clinical phenotype of the patient with *CLCN5* p.C711R mutation was less severe than that of the children with p.C160R mutation. The patient with *CLCN5 p*.L73X, 829 showed isolated protein in the early stage and a slight lesion of the glomerulus was detected by renal biopsy. This is consistent with what has been reported in the literature (Blanchard et al. 2016). This patient was treated with growth hormone at the age of three, and his height was significantly elevated (from 83 to 93 cm within 1 year).

Hypercalciuria and LMWP were noted in all of the patients with Dent disease. However, mutational analysis should be carried out in patients with these two characteristics, regardless of whether there is a stone or renal calcification (Hoopes et al. 2005).

In our center, the proportion of hypercalciuria was low, and this might be related to the strict control of calcium intake by patients. In the early stage of diagnosis or when there is a decrease in the glomerular filtration rate (GFR), most of the fasting urine calcium/creatinine is detected, which may cause an underestimation of urine calcium excretion. When available, a 24-h urinary calcium measurement was employed during the follow-up process. Most children developed hypercalciuria.

Recurrent stones, young age of onset growth retardation, LMWP, and extra-renal manifestations were uncommon in the diseases of PH, cystinuria, and hyperglycinuria. For these types of diseases, molecular diagnosis is important in determining the etiology, especially if stone analysis is not available. Thus, it is essential to develop further effective therapies to protect patients with different types of kidney failure who may undergo multiple surgeries. Some patients may also rapidly progress to ESRD.

For PH, which is indicated the AR genetic model, PH1 is the most common and most severe type, caused by mutations in the *AGXT* gene and accounting for approximately 80% of cases. PH type-II (PH2) is caused by mutations in the *GRHPR* gene, and PH3 is caused by mutations in the *HOGA* gene. Both showed a mild clinical phenotype, manifested as urolithiasis (Cochat and Rumsby 2013). In our previous work, we administered potassium citrate and hydration in patients diagnosed with PH3 to reduce their development of additional stones. In addition, Vitamin B6 therapy proved effective in patients with partial genotypes of PH1. We also administered vitamin B6 therapy in patients diagnosed with PH1, whose renal function should be followed-up in the next stage.

The two patients with a definite diagnosis of cystinuria, due to detection of variations in the *SLC3A1* and *SLC7A9* genes and stone analysis suggesting l-cystine, were treated with strict alkalization urine therapy because they were too young to develop a typical staghorn stone. Stone progression was very slow during a 2-year follow-up.

In addition to the currently known monogenic disorders, two genes and one CNV not previously identified and characterized as causative of NL/NC were detected in our cohort. They are involved in epigenetic modifications with the potential to cause mental retardation. This was relevant to three cases with Kabuki syndrome caused by *KMT2D* gene mutation, one case with *KAT6A* gene mutation, and one case with a 5.05-Mb draft genome sequence at chr10:130378377–135427935 q26.2–q26.3. This group of patients had a special manifestation, including facial dysmorphism and psychomotor retardation (Table 2, Supplemental Table 3). A summary of manifestations in this type of disease revealed that facial dysmorphism, psychomotor retardation, skeletal anomalies, and short stature are common, and CAKUT is a lot more common. All of these cardinal manifestations, especially for a facies, are absolutely contributory to the diagnosis. The causes of NL are not well understood, however, genetic testing could ultimately help confirm the diagnosis.

Kabuki syndrome, which is caused by *MLL2* or *KMT2D* mutations, has characteristic manifestations, such as facial dysmorphism, related to histone methylation. Histone methylation is an important epigenetic modification, involved in the regulation of gene expression, development, and injury response. Current reports suggest that Kabuki is mainly associated with *MLL2* mutation, which is involved in kidney function, while *KMT2D* mutation is generally less involved in kidney function. There are few previous reports of kidney involvement for *KMT2D*. However, in China, *KMT2D* is the more renal function-associated gene. This is consistent with the three patients diagnosed with mutations in *KMT2D* (Arnaud et al. 2015). However, the exact mechanism responsible for formation of stones remains elusive. Renal ultrasound showed mild renal dysplasia occurring in two of our three patients, and none of the patients had recurrent UTI.

The burden of rare CNVs is high in a significant proportion of patients with CAKUT (Verbitsky et al. 2019), however, the pathogenesis of NL in these patients is not clear. This may be related to recurrent UTI and anatomical abnormalities. The NL-related comorbidities, such as UTI, may specifically lead to progressive renal damage.

The *KAT6A* gene encodes a lysine acetyltransferase that is a member of the MYST family of proteins. It forms part of a histone acetyltransferase complex, thus regulating transcriptional activity and gene expression, and can lead to renal abnormalities and recurrent UTI. The mechanism of stone formation (Kennedy et al. 2019), assessed in the present study, remains elusive.

The results of the present study showed that patients whose age of onset was less than 2 years old and whose clinical manifestations were associated with a specific biochemical phenotype, such as growth retardation and osteoporosis, should urgently undergo genetic testing. However, due to the high cost of genetic testing, it is of great significance to propose more reliable genetic testing criteria to reduce the expected out-of-pocket expense.

**Fig. 2:**
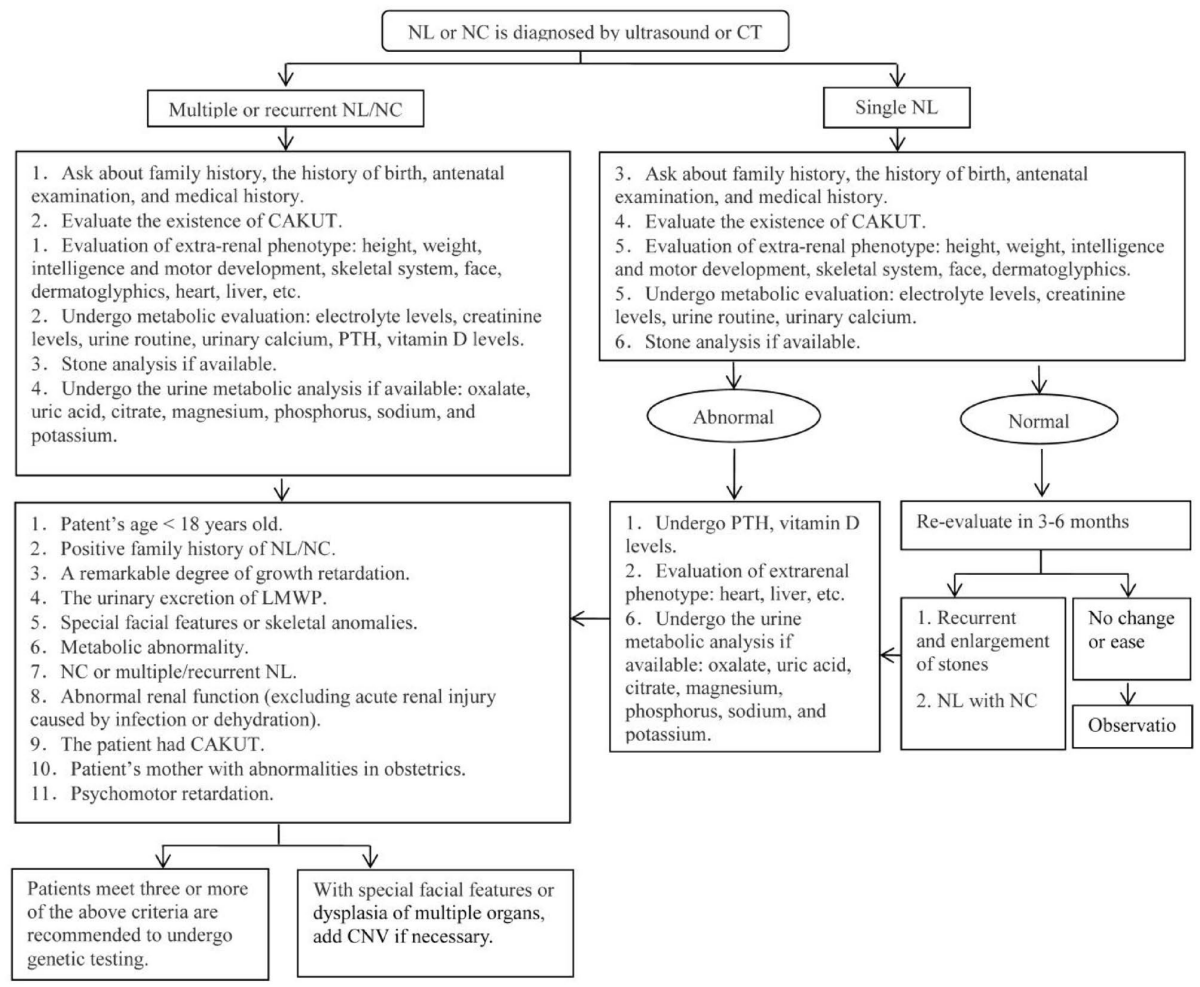
The strategy of gene testing for nephrolithiasis (NL)/nephrocalcinosis (NC) patients in our center: A flow chart showing the metabolic evaluation and the standard of gene testing of NL/NC

Patients with NL/NC who meet three or more of the following criteria were recommended to undergo genetic testing in our center. The formulation of criteria takes into consideration the results of some multicenter cohort studies and the review of the existing literature (Daga et al. 2018; Hernandez et al. 2015; Shu et al. 2017; Thongprayoon et al. 2020), as well as the reality of the economic burden (Fig. 2). These criteria include: (1) Patent’s age (< 18 years old); (2) A positive family history of NL/NC; (3) A remarkable degree of growth retardation; (4) The urinary excretion of low-molecular weight protein (LMWP) (urinary β2-microglobulin and α1-microglobulin); (5) Special facial features or skeletal anomalies; (6) Metabolic acidosis, metabolic alkalosis, or abnormal serum levels of electrolytes (excluding diarrhea, vomiting, etc.); (7) Diagnosis of NC or multiple/recurrent NL; (8) Abnormal renal function (excluding AKI caused by infection or dehydration); (9) Congenital anomalies of the kidney and urinary tract (CAKUT); (10) A mother with abnormalities in obstetrics; (11) Psychomotor retardation. The chance of detecting a genetic diagnosis was highest for patients with a specific biochemical phenotype and extrarenal phenotype. Thus, our results suggest that when hereditary pediatric NL/NC is suspected, a molecular diagnosis is important. In addition, the trio-WES is a promising option, and CNV should be considered if necessary. The high positive detection rate of molecular diagnostic testing in our center may decrease the number of patients who miss the follow-up. Thus, further criteria are in need of being adopted for gene testing via conducting multicenter cohort studies.

For patients who meet three or more of the mentioned inclusion criteria, it is highly essential to pay attention to the existence of genetic disorders when no pathogenic variation is found in the molecular diagnostic tests. Thus, it is advisable to strongly consider the clinical phenotype, follow-up on the family history, and use further advanced molecular diagnostic techniques, such as next-generation sequencing (NGS), CNV and WGS, if required (Groopman et al. 2018).

It is noteworthy that one case was highly suspected of having renal tubular disease, in which the initial ultrasound test found no abnormality, while the next confirmed the diagnosis of NC. This situation requires the vigilance of pediatricians and sonographers, while micro-CT imaging enables kidney visualization at a much higher definition (Turk et al. 2016).

Confirmed clinical diagnoses were available for several diseases, including dRTA, Dent disease, X-linked hypophosphatemia (XLH), infantile hypercalcaemia, and familial hypomagnesaemia with hypercalciuria and nephrocalcinosis (FHHNC). Genetic testing can help formulate a molecular diagnosis, guide treatment to slow down the progression of stones and calcification, and be used to provide genetic counseling. When the clinical phenotype is not helpful for diagnosis, but inherited renal tubular disease is highly suspected, genetic testing may be helpful. For some diseases, genetic testing can also be helpful in excluding the risk of recurrence in renal transplants (*AGXT*, or *CLDN16*, etc.).

For our cohort, in five patients, variants were defined as VUS. The genotypes and clinical phenotypes corresponded to co-segregation of the family but did not meet ACMG criterion to be classified as pathogenic or likely pathogenic. One patient with suspected Dent disease carried a missense mutation in *CLCN5*, one patient with suspected dRTA carried two compound heterozygous missense mutations in recessive gene *ATP6V1B1*, two patients with suspected Cystinuria and PH3 carried one heterozygous missense mutation and one likely pathogenic mutation in a recessive gene, and the detected CNV was also defined as a VUS. If possible, functional studies on missense variants should be scheduled for completion.

## Conclusions

In summary, our data suggest that the highest chance of detecting a genetic diagnosis was greatest in patients with a specific biochemical phenotype, CAKUT, and extrarenal phenotype. The outcome of NL/NC is variable ranging from trivial to CKD, and is easily overlooked because of its insidious onset. Because of the significant risk of recurrence and possible unfavorable prognosis, all children with NL/NC need a complete evaluation with clinical phenotype, and genetic testing needs to be performed in patients with high risk of hereditary diseases to offer genetic counseling and appropriate management. Thus, the results of molecular diagnostic testing require comprehensive and accurate information about phenotype and family history (Johnson et al. 2020), and regular monitoring of metabolic evaluation is required. Because of the expected out-of-pocket expense, it is of great significance to propose more reliable genetic testing criteria. Appropriate diagnosis and genetic testing criteria are the main focus of this study.

Our results suggest that when a clinical diagnosis can be confirmed, genetic testing can help formulate a molecular diagnosis, guide treatment and provide genetic counseling. Furthermore, genetic testing also yielded a molecular diagnosis for 17 out of 32 individuals with NL or NC of unknown etiology. Confirming a genetic diagnosis can be helpful in improving treatment methods, which also means starting appropriate treatment at early stages and eliminating or reducing long-term complications. In addition, knowledge of the genetic information can help in counseling. Our study demonstrated WES or WES plus CNV play a distinct role in diagnostic utility and patient management in the context of pediatric NL/NC.

The present study contains a number of shortcomings. First, although we aimed to recruit enough patients in this study, the sample size is relatively small. Second, difficulty in analysis of stone composition in children might influence the reliability of our findings. Fortunately, 24-h urine metabolic analysis is available to assist such questions. Thirdly, although some variants (missense mutations) did not meet the criteria presented by the ACMG guidelines, the genotypes and clinical phenotypes were consistent with co-segregation of family, suggesting that those variants need functional analysis. Thus, further long-term multicenter cohort studies with larger sample sizes are warranted to confirm our results.

## Supplementary Information

Below is the link to the electronic supplementary material.

Supplementary file1 (PDF 207 KB)
