# Supplementary material for: Genetic testing enables a precision medicine approach for nephrolithiasis and nephrocalcinosis in pediatrics: a single-center cohort
Source: Mol Genet Genomics. 2022 May 25;297(4):1049–61. doi: 10.1007/s00438-022-01897-z (PMC9250473; doi:10.1007/s00438-022-01897-z)
Supplement: Supplementary file 1 — Supplementary file1 (PDF 207 KB) [file 438_2022_1897_MOESM1_ESM.pdf]

Supplemental table 1 General clinical and biologic features of patients who identified hereditary nephrolithiasis (NL) and (or) nephrocalcinosis (NC) in our center

Patient number	Gender	Onset-age	Family history	Height [a]	Weight [a]	NL/NC	Renal function	LMWP	Hypercalciuria [b]	Serum electrolyte	CAKUT/Extra-renal phenotype	Stone analysis
1	M	2y2m	no	10th	25th	NC	N	Y	Y	N	N/PMR	NA
2	M	3y3m	no	<3rd	<3rd	NC	N	Y	Y	N	N/N	NA
3	M	4y11m	no	<3rd	<3rd	NL + NC	N	Y	Y	N	N/N	NA
4	M	14y	no	75th	75th	NC	N	Y	N	N	N/N	NA
5	M	12.5y	no	<3rd	3rd-10th	NC	CKD2	Y	N	hypokalemia	N/knock knees	NA
6	F	9m	no	<3rd	<3rd	NC	N	Y	N	hypokalemia	N/N	NA
7	M	6y	no	<3rd	<3rd	NL	N	Y	Y	hypokalemia	N/knock knees	NA
8	F	1y8m	no	3rd-10th	<3rd	NC	N	Y	N	hypokalemia	N/N	NA
9	F	3m	no	<3rd	<3rd	NC	N	N	N	hypokalemia	N/ASD	NA
10	M	3y11m	no	<3rd	<3rd	NL + NC	N	Y	Y	hypokalemia	N/N	NA
11	F	3y9m	Maternal NL	<3rd	3rd-10th	NC	N	N	N	hypophosphatemia	N/bow legs, PMR	NA
12	F	2y11m	Maternal NL	50th	25th	NC	N	N	N	hypomagnesemia	N/N	NA
13	F	11m	no	10th-25th	<3rd	NC	CKD2	N	Y	hypercalcemia	N/muscle weakness, PMR	NA
14	M	10m	no	3rd	25th	NL	N	N	N	N	N/ASD	L-cystine stone
15	M	6y7m	no	25th	75th	NL	N	N	N	N	N/N	L-cystine stone
16	M	4m	no	90th	25th	NL + NC	N	N	N	N	N/renal rickets	NA
17	M	11m	no	3rd-10th	50th	NL	N	N	N	N	N/N	CaOx
18	M	13m	no	<3rd	25th	NL	N	Y	Y	N	N/N	CaOx
19	M	6m	Maternal NL	90th	90th	NL	N	N	N	N	N/PMR, ASD	NA
20	M	9m	no	3rd-10th	3rd-10th	NL	N	N	N	N	N/facial dysmorphism, PMR, ASD	NA
21	M	1y8m	no	<3rd	<3rd	NL	N	Y	NA	N	N/facial dysmorphism, PMR , ASD+VSD+PH	NA

22	M	8y	no	<3rd	10th	NC	N	Y	Y	N	N/facial dysmorphism, PMR, VSD	NA
23	M	3m	no	<3rd	<3rd	NL signal	N	Y	N	N	VUR + NB/facial dysmorphism, PMR	NA
24	M	8y	no	50th	25th-50th	NL + NC	N	N	N	N	renal cyst, ASD	NA
25	M	3m	no	<3rd	<3rd	NL + NC	CKD2	NA	N	N	N/facial dysmorphism, PMR, ASD	NA

a: The height and weight value is according to the percentage value table of body height and body mass of Chinese children and adolescents. b: Normal urinary values in spot urine samples and normal urinary values in 24-Hour urine collection: urine calcium-to-creatinine ratio: <7 mo <0.86, 7-18 mo <0.60, 19 mo-6 y <0.42, >6 y <0.20 (mg/mg) or 24-hour urinary calcium <4 mg/kg/24h; N: normal; Y:yes; NA: Not available; F: famale; M: male; LMWP: low-molecular weight protein (the elevated levels of urinary β2-microglobulin and α1-microglobulin); CAKUT: congenital anomalies of the kidney and urinary tract; PH: pulmonary hypertension; ASD: atrial septal defect; VSD: Ventricular septal defect; CKD: chronic kidney disease; VUR: vesicoureteric reflux; NB: neurogenic bladder; PMR: psychomotor retardation;

Supplemental table 2 General clinical and biologic features of patients who identified nephrolithiasis (NL) and (or) nephrocalcinosis (NC) but with negative genetic analysis in our center

Patient number	Gender	Onset-age	Family history	Height a	Weight a	NL/NC	Renal function	LMWP	Hypercalciuria b	Serum electrolyte	CAKUT/ Extra-renal phenotype	Stone analysis
1	F	36m	no	<3rd	10th	NL	N	N	N	N	N/PMR	NA
2	F	35m	yes	75th	75th	NC	N	N	N	N	N/N	NA
3	M	22m	no	50th	10th	NL single	N	N	N	N	N/ASD	NA
4	M	12m	yes	10th	10th	NC	N	N	N	N	N/N	NA
5	F	7y	yes	50th	10th	NL	CKD2	Y	N	N	N/N	NA
6	M	38m	yes	50th	50th	NL single	N	N	N	N	N/N	NA
7	F	4.5y	yes	10th-25th	25th-50th	NL + NC	N	N	N	N	hydronephrosis	NA

Supplemental table 3 Outcome of genetic testing in 25 out of 32 NL and (or) NC patients with positive genetic variants

Patient	Gene	Nucleotide change	Amino acid change	Segregation	Mode	NL / NC	ACMG criteria	ACMG Evidence	PMID Ref.	Genetic diagnosis OMIM	OMIM
**Genes of failed urinary acidification and hypercalciuria**											
1	*OCRL1*	c.556 A > T (NM_000276)	p.K186X , 716	Maternal	XLR	NC	Pathogenic	PVS1+PM1+PM2+PP3	Novel	DD2	300535

										300555	
2	OCRL1	c.218 T > A (NM_000276)	p.L73X , 829	Maternal	XLR	NC	Pathogenic	PVS1+PM1+PM2+PP3	Novel	DD2<br>300555	300535
3	*CLCN5*	c.478 T > C (NM_001127899)	p.C160R	Maternal	XLR	NL + NC	Uncertain (VUS)	PM2+PP3+PP1	Novel	DD1<br>300009	300008
4	*CLCN5*	c.2131 T > C (NM_000084)	p.C711R	Maternal	XLR	NC	Likely pathogenic	PM1+PM2+PM5+PP3	Novel	DD1<br>300009	300008
5	*SLC4A1*	c.2102 G > A (NM_000342)	p .G701D	Hom	AR	NC	Pathogenic	PS+PS1+PS4+PM1+PM2+PM	9854053 Ref. 1	dRTA<br>611590	109270
6	*SLC4A1*	c.1765 C > T (NM_000342)	p.R589C	de novo	AD	NC	Pathogenic	PS2+PS1+PM1+PM2+PP3	9312167 Ref. 2	dRTA<br>179800	109270
7	*SLC4A1*	c.1766 G > A (NM_000342)	p.R589H	de novo	AD	NL	Pathogenic	PS2+PS1+PM1+PM2+PP3	9312167 Ref. 2	dRTA<br>179800	109270
8	*ATP6V1B1*	c.1153 C > A (NM_001692)<br>c.806 C > T (NM_001692)	p.P385T<br>p .P269L	Paternal<br>Maternal	AR	NC	Uncertain (VUS)<br>Uncertain (VUS)	PM1+PM2+PP3<br>PM1+PM2+PP3	Novel<br>Novel	dRTA<br>267300	192132
9	*ATP6V0A4*	c.1899 C ＞ A (NM_020632)	p.Y633X , 208	Hom	AR	NC	Pathogenic	PVS1+PM2+PP3	Novel	dRTA<br>602722	605239
10	*SLC4A1*	c.1765 C > T (NM_000342)	p.R589C	de novo	AD	NL + NC	Pathogenic	PS2+PS1+PM1+PM2+PP3	9312167 Ref. 2	dRTA<br>179800	109270
11	*PHEX*	c.1735 G > A (NM_000444)	p.G579R	Maternal	XLD	NC	Pathogenic	PS2+PS1+PM1+PM2+PP3	9097956 Ref. 3	XLH<br>307800	300550
12	*CLDN16*	loss1 (exon: 3) (NM_006580.3)<br>c.427 + 5 (IVS2) G > A (NM_006580)	166 bp<br>-	Paternal<br>Maternal	AR	NC	Likely Pathogenic<br>Likely Pathogenic	PVS1+PM2<br>PM2+PP5+PP3	Novel<br>25852890 Ref. 4	FHHNC<br>248250	603959
13	*CYP24A1*	c.1310 C > A (NM_000782)	p.P437H	Hom	AR	NC	Likely pathogenic	PM1+PM2+PP3	30633617 Ref. 5	IH<br>143880	126065

Genes of hyperuricosuria, cystinuria, hyperglycinuria

14	*SLC3A1*	c.541C > T (NM_000341)<br>c.850 G > C (NM_000341)<br>c.1173 C > G (NM_000341)	p.R181W<br>p.D284H<br>p.D391E	Paternal<br>Maternal<br>Maternal	AR	NL	Likely Pathogenic<br>Likely pathogenic<br>Uncertain (VUS)	PM1+PM2+PM<br>PM1+PM2+PM<br>PM1+PM2+PP3	Novel<br>Novel<br>Novel	Cystinuria<br>220100	104614
15	*SLC7A9*	c.376 G > A (NM_001243036)<br>c.149 T > G (NM_001243036)	p.A126T<br>p.V50G	Paternal<br>Maternal	AR	NL	Likely pathogenic<br>Uncertain (VUS)	PS1+PM2+PP3<br>PM1+PM2+PP3	1115779 Ref. 6<br>Novel	Cystinuria<br>220100	604144
16	*AGXT*	c.25 _ c.26 insC (NM_000030)<br>c.121 G > A (NM_000030)	p.T9Tfs * 159<br>p.G41R	Paternal<br>Maternal	AR	NL + NC	Pathogenic<br>Pathogenic	PVS1+PM3+PP5<br>PS1+PM1+PM2+PP2+PP3	Novel<br>8101040 Ref. 7	PH1<br>259900	604285
17	*HOGA1*	c.834 + 1 (IVS6) G > T (NM_138413）<br>c.356 T > G (NM_138413）	-<br>p.V119G	Paternal<br>Maternal	AR	NL	Pathogenic<br>Likely pathogenic	PVS1+PS1+PM2+PP3<br>PM3+PM1+PM2+PP3	25972204 Ref. 8<br>Novel	PH3<br>613616	613597
18	*HOGA1*	c.103 A > G (NM_138413)<br>c.845 G > A (NM_138413)	p.I35V<br>p.R282H	Paternal<br>Maternal	AR	NL	Uncertain (VUS)<br>Likely pathogenic	PM1+PM2<br>PS4 +PM1+PM2+PP3	Novel<br>Novel	PH3<br>613616	613597

19	*SLC6A19*	c.284 G > C(NM_001003841)	p.R95P	Paternal	AR	NL	Likely pathogenic	PM1+PM2+PP3+PP1	Novel	FG	608893
		c.461 C > T(NM_001003841)	p.P154L	Maternal			Likely pathogenic	PM1+PM2+PP3+PP1	Novel	167030	
		c.1018 C > T (NM_213613)	p.R340C	Maternal	AR/AD		SNP	-	-		610130
	SLC26A1										

Other genes

20	*KMT2D*	c.15113_c.15115 del AGG (NM_003482)	p.E5038_G5039delinsG	de novo	AD	NL	Likely pathogenic	PS2+PM2+PM4	Novel	KS 147920	602113
21	*KMT2D*	c.6595 delT (NM_003482)	p.Y2199fs	de novo	AD	NL	Pathogenic	PVS1+PS2+PS1+PM1+PM2+PP3	Novel	KS 147920	602113
22	*KMT2D*	C.15686 dupG (NM_003482)	P.C5230Lfs*5	de novo	AD	NC	Pathogenic	PVS1+PS2+PS1+PM1+PM2+PP3	Novel	Ks 147920	602113
23	*KAT6A*	c.3070 C > T (NM_006766)	p.R1024X , 981	de novo	AD	NL signal	Pathogenic	PVS1+PS2+PS1+PM2+PP3	Novel	ATS 616268	601408
24	*PKD1*	c.9806 G > A (NM_001009944)	p.R3269Q	Maternal	AD	NL + NC	Likely pathogenic	PM1+PM2+PP3	Novel	ADPKD 173900	601313
25	CNV	chr10: 130378377 - 135427935 q26.2 - q26.3	-	de novo	-	NL + NC	VUS	The parents are wild type and have no clinical phenotype. There is no report in DGV ordinary people database. Clinvar database has partial overlap of several possible pathogenic CNV fragments involved in renal dysplasia.	Novel	CNV	

ACMG: American College of Medical Genetics and Genomics; PMID: PubMed ID; Hom: homozygous mutation; AD: autosomal dominant inheritance; AR: autosomal recessive inheritance; XLR: X-linked recessive inheritance; XLD: X-linked dominant inheritance; CNV: chromosome copy number variation; SNP: single nucleotide polymorphism; DD: Dent disease; XLH: X-1inked dominant hypophosphate; dRTA: distal renal tubular acidosis; IH: infantile hypercalcaemia; FHHNC: Familial hypomagnesaemia with hypercalciuria and nephrocalcinosis; PH: primary hyperoxaluria; FG: familial glycosuria; KS: Kabuki syndrome; ADPKD: autosomal dominant polycystic kidney disease; ATS: Arboleda-Tham syndrome; VUS: variant of unknown significance.

Analysis of new missense mutations in pathogenicity analysis using multiple protein function prediction software PP2 (polyphen2 http://genetics.bwh.harvard.edu/pph2/) and SIFT (sorting intolerant from tolerant http://sift.jcvi.org/ MutationTaster: http://www.mutationtaster.org).

Ref:

1. Rowe PS, Oudet CL, Francis F, et al. Distribution of mutations in the PEX gene in families with X-linked hypophosphataemic rickets (HYP). Hum Mol Genet. 1997;6(4):539-549. doi:10.1093/hmg/6.4.539.

2. Bruce LJ, Cope DL, Jones GK, et al. Familial distal renal tubular acidosis is associated with mutations in the red cell anion exchanger (Band 3, AE1) gene. J Clin Invest. 1997;100(7):1693-1707. doi:10.1172/JCI119694.

3. Tanphaichitr VS, Sumboonnanonda A, Ideguchi H, et al. Novel AE1 mutations in recessive distal renal tubular acidosis. Loss-of-function is rescued by glycophorin A. J Clin Invest. 1998;102(12):2173-2179. doi:10.1172/JCI4836.

4. Hanssen O, Castermans E, Bovy C, et al. Two novel mutations of the CLDN16 gene cause familial hypomagnesaemia with hypercalciuria and nephrocalcinosis. Clin Kidney J. 2014;7(3):282-285. doi:10.1093/ckj/sfu019.

5. Sun Y, Shen J, Hu X, et al. CYP24A1 Variants in Two Chinese Patients with Idiopathic Infantile Hypercalcemia. Fetal Pediatr Pathol. 2019;38(1):44-56. doi:10.1080/15513815.2018.1492052.

6. Tostivint I, Royer N, Nicolas M, et al. Spectrum of mutations in cystinuria patients presenting with prenatal hyperechoic colon. Clin Genet. 2017;92(6):632-638. doi:10.1111/cge.13079.

7.Danpure CJ, Purdue PE, Fryer P, et al. Enzymological and mutational analysis of a complex primary hyperoxaluria type 1 phenotype involving alanine:glyoxylate aminotransferase peroxisome-to-mitochondrion mistargeting and intraperoxisomal aggregation. Am J Hum Genet. 1993;53(2):417-432.

8.Allard L, Cochat P, Leclerc AL, et al. Renal function can be impaired in children with primary hyperoxaluria type 3. Pediatr Nephrol. 2015;30(10):1807-1813. doi:10.1007/s00467-015-3090-x.
